# Equity in the delivery of community healthcare to older people: findings from 10/66 Dementia Research Group cross-sectional surveys in Latin America, China, India and Nigeria

**DOI:** 10.1186/1472-6963-11-153

**Published:** 2011-06-28

**Authors:** Emiliano Albanese, Zhaorui Liu, Daisy Acosta, Mariella Guerra, Yueqin Huang, KS Jacob, Ivonne Z Jimenez-Velazquez, Juan J Llibre Rodriguez, Aquiles Salas, Ana L Sosa, Richard Uwakwe, Joseph D Williams, Guilherme Borges, AT Jotheeswaran, Milagros G Klibanski, Paul McCrone, Cleusa P Ferri, Martin J Prince

**Affiliations:** 1 King's College London, Institute of Psychiatry, Health Services and Population Research Department, London, UK; 2 Peking University, Institute of Mental Health. Beijing, China; 3 Universidad Nacional Pedro Henriquez Ureña (UNPHU), Internal Medicine Department, Geriatric Section, Santo Domingo, Dominican Republic; 4 Psychogeriatric Unit, National Institute of Mental Health "Honorio Delgado Hideyo Noguchi", Lima, Perú; 5 Christian Medical College, Vellore, India; 6 Internal Medicine Dept., Geriatrics Program, School of Medicine, Medical Sciences Campus, University of Puerto Rico, San Juan, Puerto Rico; 7 Facultad de Medicina Finley-Albarran, Medical University of Havana, Havana, Cuba; 8 Medicine Department, Caracas University Hospital, Faculty of Medicine, Universidad Central de Venezuela, Caracas; 9 The Cognition and Behavior Unit, National Institute of Neurology and Neurosurgery of Mexico, Mexico City, Mexico; 10 Nnamdi Azikiwe University Teaching Hospital, Nnewi, Anambra State, Nigeria; 11 Department of Community Health, Voluntary Health Services, Chennai, India; 12 Instituto Nacional de Psiquiatria, Mexico City, Mexico, and the Universidad Autonoma Metropolitana, Mexico City, Mexico; 13 Public Health Foundation of India, Delhi, India and King's College London, Institute of Psychiatry, Health Services and Population Research Department, London, UK; 14 Policlinico 14 de Junio. Luyano. Municipio 10 de Octubre, Havana, Cuba

## Abstract

**Background:**

To describe patterns of recent health service utilisation, and consequent out-of-pocket expenses among older people in countries with low and middle incomes, and to assess the equity with which services are accessed and delivered.

**Methods:**

17,944 people aged 65 years and over were assessed in one-phase population-based cross-sectional surveys in geographically-defined catchment areas in nine countries - urban and rural sites in China, India, Mexico and Peru, urban sites in Cuba, Dominican Republic, Puerto Rico and Venezuela, and a rural site in Nigeria. The main outcome was use of community health care services in the past 3 months. Independent associations were estimated with indicators of need (dementia, depression, physical impairments), predisposing factors (age, sex, and education), and enabling factors (household assets, pension receipt and health insurance) using Poisson regression to generate prevalence ratios and fixed effects meta-analysis to combine them.

**Results:**

The proportion using healthcare services varied from 6% to 82% among sites. Number of physical impairments (pooled prevalence ratio 1.37, 95% CI 1.26-1.49) and ICD-10 depressive episode (pooled PR 1.21, 95% CI 1.07-1.38) were associated with service use, but dementia was inversely associated (pooled PR 0.93, 95% CI 0.90-0.97). Other correlates were female sex, higher education, more household assets, receiving a pension, and health insurance. Standardisation for age, sex, physical impairments, depression and dementia did not explain variation in service use. There was a strong borderline significant ecological correlation between the proportion of consultations requiring out-of-pocket costs and the prevalence of health service use (r = -0.50, p = 0.09).

**Conclusions:**

While there was little evidence of ageism, inequity was apparent in the independent enabling effects of education and health insurance cover, the latter particularly in sites where out-of-pocket expenses were common, and private health insurance an important component of healthcare financing. Variation in service use among sites was most plausibly accounted for by stark differences in the extent of out-of-pocket expenses, and the ability of older people and their families to afford them. Health systems that finance medical services through out-of-pocket payments risk excluding the poorest older people, those without a secure regular income, and the uninsured.

## Background

Sharp increases are forecast in the burden of chronic diseases in low and middle income countries (LMIC), as a result of demographic ageing, and the health transition [1]. In 2005, the WHO announced an ambitious goal to reduce chronic disease deaths by 2% per year [2], considered achievable through population prevention strategies targeting dietary salt and tobacco use [3]. The control of chronic diseases requires functioning, affordable and equitable primary healthcare since it is through access to these services that those at high risk can be identified, advised and treated [4]. Unfortunately, healthcare systems and services in LMIC are often unsuited to the needs of their ageing populations [5]. Structural reforms introduced fees for service, necessitating substantial out-of-pocket payments. Those with chronic diseases needing continuous treatment over long periods, and older people with little or no personal income are particularly affected. A paradigm shift has been called for, from preoccupation with simple curative interventions to chronic disease management, long-term support and care. Given the frailty of many older people there is also a need for outreach, assessing and managing patients in their own homes [4,6].

The notion of equity in health care is predicated on principles of social justice underpinned by the view that access to health care should be based upon 'need' while financing should be determined according to 'ability to pay' [7]. The right to healthcare is an essential component of the universal right to health, enshrined in Article 25 (1) of the Universal Declaration of Human Rights (1948) [8], and underlined in Article 1 of the UN Convention on the Rights of Persons with Disabilities (2006) [9]. Paragraphs 74-77 of the Madrid International Plan of Action on Ageing (2002) call for the elimination of social and economic inequalities in access to healthcare and the development of healthcare and long-term care to meet the needs of older persons [10]. However, there have been very few studies of healthcare utilization among older people in LMIC, and surprisingly little focus on equity. In a large cross-sectional survey in India of those aged 60 years and over, socioeconomic status was inversely associated with ill health but positively associated with hospitalisation in the previous year in both sexes, after controlling for health status, age and social support [11]. In surveys in 10 countries in Latin America and the Caribbean [12]; health service utilisation increased with household expenditure among those aged 65 years and over for all countries other than Argentina and Chile. We were unable to find any information on the impact of health insurance coverage on older people in countries with low and middle incomes, but in the USA Health and Retirement Survey, women aged 55 to 64 years of age were two to seven times more likely to use health services for which they had insurance cover [13]. Much research has focussed upon the effects of gender on healthcare utilization among older people. Older women tend to use primary care and community health services more than men [14,15]. Gendered differences in illness constructions, help-seeking behaviour, and accessibility of services have been suggested as mediating factors [14,15]. Some investigators have discerned a 'health disadvantage' for older women, but this depends on the health outcome studied. Men are more likely to use emergency services, and as likely if not more likely to be hospitalised [11,14], and have a shorter life expectancy and a higher mortality than women in late life [16].

The aims of this study are:

1. To describe patterns of recent health service utilisation, and consequent out-of-pocket expenses in LMIC, among community-dwelling people aged 65 years and over;

2. To assess the equity with which services are accessed and delivered. Determinants of service use are often categorised into predisposing, enabling and need variables. Associations with predisposing and enabling factors, having controlled for need, indicate potential inequity. We sought to determine if younger age, female gender, higher education, higher socioeconomic status, and the absence of mobility restrictions are associated with health service utilisation after controlling for health status as an index of the need for healthcare.

## Methods

### Participants and procedures

One-phase population-based surveys were carried out, between 2003 and 2005, of all older people aged 65 years and over living in geographically defined catchment areas in nine countries - urban and rural sites in China, India, Mexico and Peru, urban sites in Cuba, Dominican Republic, Puerto Rico and Venezuela, and a rural site in Nigeria [17]. Catchment area sites were selected purposively with a view to identifying typical central urban high density, and predominately low socioeconomic status districts in national or state capital cities (Beijing in China, Chennai in India, Havana and neighbouring Matanzas in Cuba, Santo Domingo in Dominican Republic, Caracas in Venezuela, Mexico City in Mexico, Lima in Peru and Bayamón in Puerto Rico) and contrasting rural areas, with a traditional agrarian lifestyle and low-density population in the five countries where this was feasible (villages around Vellore in India, Daxing in China, Morelos state in Mexico, Canete in Peru, and Anambra state in Nigeria. The boundaries of each catchment area were precisely defined, and households mapped. Each household was then systematically door-knocked to identify all those aged 65 years and over, who were then considered eligible for participation in the survey. The 10/66 Dementia Research Group (DRG) baseline survey protocol comprises a participant questionnaire, a structured clinical interview, an informant interview, and a physical examination, and generates information regarding demographic characteristics, physical health, dementia diagnosis, mental disorders, chronic diseases risk factors, disability, health service utilisation, care arrangements and caregiver strain [17]. The target sample size for each country was between 2000 and 3000, and 1000 in Nigeria. Recruitment was on the basis of informed signed consent. Studies were approved by local ethical committees in each country and by the ethical committee of the Institute of Psychiatry, King's College London.

## Measures

Only the assessments relevant to the current analyses of the correlates of the use of community healthcare services will be described in detail here.

A. Determinants of healthcare use, comprising assessments of predisposing, enabling and need variables

1) Participants' sociodemographic characteristics (predisposing variables). Age, sex, educational level (no education, some, completed primary, completed secondary, completed tertiary), marital status (currently married versus never married, widowed or separated) and co-residence with children.

2) Socioeconomic status (enabling variables). Wealth was assessed according to the number of reported household assets (motor vehicles; television; fridge and/or freezer; water and electricity utilities; telephone; plumbed toilet; plumbed bathroom). Participants were asked to disclose whether they received income from an occupational or government pension, and whether they had purchased any medical insurance cover.

3) Health status (need variables). Physical health was assessed through self-report of a list of 11 commonly occurring physical impairments [18]; paralysis, weakness or loss of a limb; eyesight problems; stomach or intestine problems; arthritis or rheumatism; heart problems; hypertension; hearing difficulties or deafness; breathlessness; difficulty breathing or asthma; faint or blackouts; skin disorders; persistent cough, which were coded as present if they interfered with activities 'a little' or 'a lot'. Dementia diagnosis, meeting criteria for either or both of the cross-culturally validated 10/66 algorithm [19] or DSM-IV dementia [20]. Mild, moderate or severe depressive episode according to the International Classification of Diseases tenth edition [21] ascertained using a structured clinical interview, the Geriatric Mental State Examination [22]. Restricted mobility was assessed using the question "How much difficulty did you have in walking a long distance, such as a kilometer?" Those reporting severe or extreme difficulty were identified as having restricted mobility.

B. The outcome - healthcare utilization was determined using the Client Service Receipt Inventory [23], adapted for use in LMIC [24], covering a range of potentially relevant healthcare services: government primary care, hospital outpatients, private doctors, other community health services, traditional healers, dentists and hospital admission. The frequency of use in the last three months, and the average out-of-pocket cost of the consultations were ascertained. Given the multiplicity of primary healthcare providers, for the purposes of the current analysis use of community healthcare services was defined as any reported use in the three months prior to the survey of government primary care, hospital outpatient, private doctor, traditional healer and other community medical services.

### Statistical analyses

We describe participants' socio-demographic and health status by site, and the proportion of participants reporting use of each individual health service, and any community healthcare services over the past three months. To facilitate comparison of use of any community healthcare service among sites we carried out direct standardisation (using the entire pooled sample as the external standard population) for the effects of age group, sex, number of physical impairments, and dementia and depression diagnoses. We also describe, for each site, the proportion of those that reported using government primary care, hospital outpatient services and private doctor services that also reported some out-of-pocket expense for use of that service. By site, we used Poisson regression to calculate prevalence ratios (PRs) with robust 95% confidence intervals (CI) controlling for household clustering, for the association between use of any community healthcare services and the following variables; age group, sex, educational level, marital status, co-residence, number of household assets (dichotomised at the median in each site), health insurance, dementia diagnosis, ICD-10 depressive episode, and number of physical illnesses. The effect of mobility restriction was tested in an extension to the above model, since this might be one mechanism by which the effects of mental, physical and cognitive disorders might be mediated, and to include it in the first model might lead to underestimation of their effects. Finally we used fixed or random effect meta-analyses to combine the site-specific adjusted PRs for the associations with each of the explanatory variables. Between sites heterogeneity was formally tested (Cochran's Q) and Higgins I^2 ^values calculated (with 95% CI) [25]. Higgins I^2 ^estimates the percentage of total variation across studies attributable to heterogeneity rather than sampling error. Finally, in an exploratory analysis, we estimated, at the ecological level, the associations (Pearson's correlation coefficients) between the prevalence of health service use and the proportion of consultations requiring out-of-pocket expenses, health insurance coverage, and pension coverage. All analyses were conducted using STATA 9.2 and release 2.3 of the 10/66 dataset.

## Results

The achieved sample was 17,944 participants, with response rates over 80% in all centres other than urban China (74%) and rural India (72%) (Table 1).

**Table 1:** Participants' Socio-Demographic and Health Characteristics

Variable	Cuba	Dominican Republic	Puerto Rico	Peru urban	Peru Rural	Venezuela	Mexico urban	Mexico rural	China urban	China rural	India urban	India rural	Nigeria
**Response rate **(%)	94	95	93	80	88	80	84	86	74	96	72	98	98
**Achieved sample **(n)	2944	2011	2008	1381	552	1965	1003	1000	1160	1002	1005	999	914
**Age in years **(MV*)	7	0	0	1	0	4	1	0	0	0	4	0	0
Mean (SD)	75.1 (7.0)	75.3 (7.5)	76.3 (7.4)	75.0 (7.4)	74.2 (7.3)	72.3 (6.9)	74.5 (6.6)	74.1 (6.7)	73.9 (6.2)	72.4 (6.0)	71.3 (6.1)	72.6 (5.8)	72.7 (7.6)
**Gender **(MV)	0	2	6	0	0	33	0	0	0	0	15	0	0
Female (%)	1913 (65.0%)	1325 (65.9%)	1347 (67.3%)	888 (64.3%)	295 (53.4%)	1226 (63.5%)	666 (66.4%)	602 (60.2%)	661 (57.0%)	556 (55.5%)	571 (57.7%)	545 (54.6%)	539 (59.0%)
**Education **(MV)	8	19	9	8	8	40	2	0	0	0	2	0	6
Did not complete primary education (%)	730 (24.9%)	1414 (71.0%)	461 (23.1%)	127 (9.3%)	225 (41.3%)	601 (31.2%)	581 (58.1%)	837 (83.7%)	385 (33.2%)	693 (69.2%)	662 (66.0%)	855 (85.6%)	678 (74.2%)
**Marital status **(MV)	8	15	6	10	1	45	0	1	0	0	3	0	67
Currently married (%)	1271 (43.3%)	586 (29.4%)	967 (48.3%)	784 (57.2%)	308 (55.9%)	921 (48.0%)	470 (46.9%)	538 (53.9%)	829 (71.5%)	585 (58.4%)	523 (52.2%)	481 (48.1%)	581 (68.6%)
**Coresidence **(MV)	0	0	0	0	0	0	0	0	0	0	0	0	
Living with children (%)	1422 (48.3%)	963 (47.9%)	611 (30.4%)	890 (64.4%)	326 (59.1%)	1578 (80.3%)	565 (56.3%)	523 (52.3%)	446 (38.4%)	679 (67.8%)	719 (71.5%)	625 (62.6%)	‡
**Number of assets **(MV)	8	5	0	0	0	0	0	0	1	0	4	0	
Median (interquartile range)	6 (5-6)	5 (4-6)	7 (6-7)	6 (6-6)	5 (4-6)	6 (6-7)	6 (6-7)	4 (3-6)	5 (5-6)	6 (5-7)	4 (3-5)	3 (2-4)	‡
**Pension coverage **(MV)	0	0	0	0	0	0	0	0	0	0	0	0	0
Disclosed government and/or occupational pension (%)	2417 (82.1%)	611 (30.4%)	1051 (52.3%)	908 (65.7%)	357 (64.7%)	1147 (58.4%)	729 (72.7%)	254 (25.4%)	1050 (90.5%)	38 (3.8%)	117 (11.6%)	346 (34.6%)	9 (1.0%)
**Health Insurance coverage **(MV)		4	6	8	1	62	0	0	1	0	5	0	79
Possess health insurance (%)	n/a	430 (21.4%)	1909 (95.4%)	1111 (80.5%)	399 (72.3%)	862 (43.9%)	546 (54.4%)	280 (28.0%)	14 (1.2%)	769 (76.8%)	13 (1.3%)	0 (0.0%)	1 (0.1%)
**Dementia **(MV)	0	0	0	0	0	0	0	0	0	0	0	0	0
10/66 or DSM-IV criteria (%)	323 (11.0%)	242 (12.0%)	233 (11.6%)	130 (9.4%)	36 (6.5%)	145 (7.4%)	93 (9.3%)	87 (8.7%)	84 (7.2%)	56 (5.6%)	75 (7.5%)	108 (10.8%)	87 § (9.5%)
**Depression **(MV)	0	0	0	0	0	0	0	0	0	0	0	0	0
Any ICD-10 depressive episode (%)	144 (4.9%)	278 (13.8%)	47 (2.3%)	87 (6.3%)	16 (2.9%)	107 (5.5%)	47 (4.7%)	45 (4.5%)	3 (0.3%)	7 (0.7%)	39 (3.9%)	126 (12.6%)	5 (0.5%)
**Physical impairments **(MV)	6	2	6	1	1	33	0	0	0	0	1	0	21
Three or more (%)	292 (9.9%)	465 (23.1%)	429 (21.4%)	224 (16.2%)	40 (7.3%)	489 (24.9%)	158 (15.8%)	185 (18.5%)	208 (17.9%)	39 (3.9%)	41 (4.1%)	168 (16.8%)	10 (1.1%)
**Mobility restriction **(MV)	8	2	7	6	1	67	3	0	4	2	2	0	67
Severe difficulty or cannot walk one km (%)	546 (18.6%)	439 (21.9%)	603 (30.1%)	143 (10.4%)	30 (5.4%)	204 (10.7%)	126 (12.6%)	146 (14.6%)	119 (10.3%)	51 (5.1%)	84 (8.4%)	225 (22.5%)	64 (7.6%)

* MV = missing values;

† ICD-10 = International Classification of Diseases (10^th ^ed.)

‡ data on household composition and assets not available from the Nigerian centre

§ Impairment on two or more tests of memory (dementia diagnosis not available from Nigerian centre)

### Participants' characteristics

Age distributions were similar across sites, with Venezuelan, rural Chinese and Indian participants being slightly younger (Table 1). There was a preponderance of women in all sites. Educational level varied across countries, highest in Cuba, Puerto Rico and urban Peru, and lowest in Nigeria, rural India and rural China where the large majority of participants had little or no formal education. Around one half were currently married in most sites other than Dominican Republic (29.4%, with a high divorce rate) and urban China (71.5%). Living with children was the norm in most sites, other than urban China (38.4%) and Puerto Rico (30.4%). Fewer household assets were reported in rural compared with urban sites, particularly rural Mexico, and India. Reported health insurance coverage was high in Peru and urban Mexico (half to three-quarters or more of participants covered), and in Puerto Rico where 95.4% were covered by Medicare, modest in rural Mexico and Venezuela (a quarter to a half covered), low in Dominican Republic and negligible in India. Reported coverage was also negligible in urban China, but 76.8% reported having insurance in rural China (Table 1). Pension coverage was lowest in rural China, India, rural Mexico, and the Dominican Republic and highest in Cuba and urban China. Dementia prevalence ranged between 6.5% and 12.0%, somewhat lower in China and India than Latin America [26]. The prevalence of ICD-10 depression was negligible in China and Nigeria, and high in Dominican Republic (13.8%) and rural India (12.6%), otherwise ranging between 2.3 and 6.3%. A relatively small proportion of participants reported three or more physical impairments in rural Peru, rural China, urban India and Nigeria. The same pattern was observed for self-reported restricted mobility.

### Healthcare service utilization

Proportions of participants reporting use of any community healthcare services (primary care doctor, hospital-based doctor, private doctor, traditional healer and other community services) varied between 6.1% and 81.9% across sites, with particularly high levels of use in Puerto Rico (81.9%) and low levels of use in rural China (6.1%), urban China (38.6%), rural Peru (28.1%) and Nigeria (29.8%) (Table 2). In other sites, approximately one half to two-thirds had used at least one service in the past three months. The variation between sites was reduced somewhat after direct standardization for age, gender, physical impairments, depression and dementia, but the same sites stood out as having low levels of service use. Government primary care services were little used relative to other sectors in the Puerto Rico, Dominican Republic, Peru, and urban India. Private doctors were the main providers in Puerto Rico, India, and, to a lesser extent, in the Dominican Republic and Venezuela. Hospital outpatients were an important source of healthcare in urban Latin American sites, urban China and rural India. Traditional healers were little used in all sites other than rural India and Nigeria.

**Table 2:** Use of Healthcare Services, by study site

		Community Healthcare Services								
				
Centres	n	Gov't Primary Care	Hospital outpatient	Private Doctor	Other Services	Traditional Healer	Any Community Medical Service*		Hospital Admission	Dentist
							
							Crude prevalence	Standardised^† ^prevalence (95% CI)		
Cuba MV† = 6	2944	1012 (34.4%)	659 (22.4%)	4 (0.1%)	189 (6.4%)	13 (0.4%)	1465 (49.9%)	50.4% (48.7-52.2%)	62 (2.1%)	181 (6.2%)
Dominican Republic MV = 2	2011	222 (11.0%)	453 (22.5%)	409 (20.4%)	35 (1.7%)	5 (0.3%)	960 (47.8%)	45.0% (42.7-47.2%)	61 (3.0%)	87 (4.3%)
Puerto Rico MV = 6	2008	198 (9.9%)	20 (1.0%)	1461 (72.8%)	8 (0.4%)	0 (0.0%)	1639 (81.9%)	79.8% (78.0-81.6%)	107 (5.3%)	320 (16.0%)
Peru urban MV = 1	1381	155 (11.2%)	451 (32.7%)	147 (10.7%)	39 (2.8%)	5 (0.4%)	670 (48.6%)	48.1% (45.6-50.6%)	31 (2.2%)	90 (6.5%)
Peru rural MV = 1	552	66 (12.0%)	90 (16.3%)	13 (2.4%)	5 (0.9%)	5 (0.9%)	155 (28.1%)	29.3% (25.7-32.9%)	3 (0.5%)	12 (2.2%)
Venezuela MV = 33	1965	443 (22.9%)	450 (23.3%)	574 (29.7%)	147 (7.6%)	14 (0.7%)	1211 (62.7%)	60.3% (58.1-62.5%)	77 (4.0%)	156 (8.1%)
Mexico urban MV = 0	1003	362 (36.1%)	259 (25.8%)	197 (19.6%)	60 (6.0%)	17 (1.7%)	721 (71.9%)	70.2% (67.5-72.9%)	22 (2.2%)	118 (11.8%)
Mexico rural MV = 0	1000	410 (41.0%)	111 (11.1%)	186 (18.6%)	45 (4.5%)	33 (3.3%)	646 (64.6%)	63.3% (60.4-66.2%)	16 (1.6%)	36 (3.6%)
China urban MV = 0	1160	242 (20.9%)	271 (23.4%)	1 (0.1%)	2 (0.2%)	3 (0.3%)	448 (38.6%)	32.4% (29.9-34.8%)	28 (2.4%)	13 (1.1%)
China rural MV = 0	1002	38 (3.8%)	22 (2.2%)	3 (0.3%)	1 (0.1%)	0 (0.0%)	61 (6.1%)	8.7% (6.3-11.1%)	5 (0.5%)	0 (0.0%)
India urban MV = 1	1005	42 (4.2%)	124 (12.3%)	417 (41.5%)	8 (0.8%)	3 (0.3%)	566 (56.4%)	61.5% (58.7-64.3%)	10 (1.0%)	3 (0.3%)
India rural MV = 0	999	185 (18.5%)	307 (30.7%)	473 (47.3%)	185 (18.5%)	82 (8.2%)	677 (67.8%)	61.1% (57.5-64.7%)	17 (1.7%)	86 (8.6%)
Nigeria MV = 20	914	25 (2.7%)	108 (12.1%)	75 (8.4%)	41 (4.6%)	83 (9.3%)	272 (30.4%)	42.5% (37.9-47.2%)	40 (4.5%)	6 (0.7%)

* Defined as any reported use in the three months prior to the survey of government primary care, hospital outpatient, private doctor, traditional healer and other community medical services

^† ^Direct standardisation (using the entire pooled sample as the external standard population) for the effects of age group, sex, number of physical impairments, and dementia and depression diagnoses

† MV = Number of missing values

Out-of-pocket payments were required for most private doctor consultations in all sites other than Puerto Rico (Table 3). In Cuba, government primary care and hospital outpatient services were free at the point of delivery, while in China almost all consultations required out-of-pocket payments. Relatively high proportions reporting out-of-pocket payments were seen for primary care in the Dominican Republic, Peru, rural Mexico, urban India and Nigeria, and for hospital outpatients in the Dominican Republic, Mexico, rural India and Nigeria.

**Table 3:** The Proportion of Those Using Government Primary Care, Hospital Outpatients and Private Doctor Services in the Last Three Months That Reported Having Incurred Any Out-Of-Pocket Expenses For Use Of That Service, by Site And Service

Sites	Government Primary Care	Hospital outpatient	Private Doctor	Weighted proportion of consultations requiring out of pocket payments
Cuba	6/1012 (0.6%)	8/659 (1.3%)	n/a	0.8%
Dominican Republic	61/222 (27.5%)	185/453 (40.8%)	303/409 (74.1%)	50.6%
Puerto Rico	13/198 (6.6%)	0/20 (0.0%)	150/1461 (10.3%)	9.7%
Peru urban	45/155 (29.0%)	44/450 (9.8%)	100/147 (68.0%)	26.5%
Peru rural	44/66 (66.7%)	15/90 (16.7%)	10/13 (76.9%)	40.8%
Venezuela	25/443 (5.6%)	13/450 (2.9%)	389/574 (67.8%)	29.1%
Mexico urban	57/362 (15.7%)	53/259 (20.5%)	177/197 (89.8%)	35.1%
Mexico rural	123/410 (30.0%)	23/111 (20.7%)	171/186 (91.9%)	44.8%
China urban	240/242 (99.2%)	269/271 (99.3%)	1/1 (100%)	99.2%
China rural	38/38 (100%)	22/22 (100%)	3/3 (100%)	100.0%
India urban	11/41 (26.2%)	16/124 (12.9%)	403/417 (96.6%)	73.9%
India rural	20/185 (10.8%)	84/307 (27.4%)	457/473 (96.6%)	58.1%
Nigeria	11/25 (44.0%)	72/108 (66.6%)	41/75 (54.7%)	59.6%

### Correlates of use of community healthcare services

The mutually adjusted effects of participants' sociodemographic, socioeconomic and health characteristics on use of any community healthcare service are reported for each site in Table 4 model 1. Female sex and higher levels of education were overall associated with a higher prevalence of use of community health services in the past three months. However, controlling for the same covariates, men were consistently more likely to be admitted to hospital (meta-analysed PR 1.33, 95% CI 1.10-1.62, Cochrane's Q 14.1, 11 degrees of freedom, p = 0.23). There was no evidence, overall, to support an association between age, marital status, co-residence with children, and use of any community healthcare service. There was a strong association between being currently married and a higher prevalence of service use in urban India alone. The number of physical impairments, and ICD-10 depression were strongly positively associated with service use, but with considerable heterogeneity between sites. The inverse association between dementia and health service use was more consistent. Health insurance cover was positively associated with service use in most sites, but with much heterogeneity; the association was strongest in Puerto Rico, urban Peru and in China, and weakest in urban India, where only 1.3% of participants were covered. Household assets were positively associated with service use in the Dominican Republic, Puerto Rico, urban China and urban India, but the trend of the association was in the opposite direction in Cuba, rural China and rural India. In a post hoc analysis, when disclosing receipt of a government or occupational pension was substituted for household assets in the model, the effect was significant and more consistent across sites (PR 1.09, 95% CI 1.04-1.14; Cochrane's Q = 23.6 (12 df), p = 0.02). In the final stage, we added restricted mobility (extreme difficulty or incapability of walking one kilometre) to the model (Table 4 model 2). While the pooled estimate suggested no effect, again there was considerable heterogeneity, with an inverse association with service use in Cuba, and positive associations in Peru, urban Mexico and rural China.

**Table 4:** Mutually-Adjusted Prevalence Ratios (95% Confidence Intervals) For the Associations between Community Health Service Use and Socio-Demographic and Health Characteristics by Study Site

	Model 1 *										**Model 2**†
**Site**	**Age (per 5 year band)**	**Male Gender**	**Education (per level)**	**Currently Married**	**Living with children**	**Assets**	**Health** **insurance**	**ICD-10** **Depression**	**Dementia**	**Physical impairment**	**Mobility restriction**

**Cuba**	0.97 (0.94-1.01)	0.93 (0.86-1.01)	1.06 (1.02-1.10)	1.09 (1.00-1.18)	1.05 (0.98-1.13)	0.91 (0.84-0.98)	Not applicable	1.11 (0.96-1.28)	0.87 (0.76-0.98)	1.23 (1.16-1.30)	0.83 (0.74-0.92)
**Dominican** **Republic**	1.00 (0.96-1.04)	0.96 (0.87-1.07)	1.03 (0.98-1.08)	1.00 (0.90-1.12)	0.98 (0.89-1.07)	1.11 (1.01-1.22)	1.19 (1.07-1.32)	1.03 (0.91-1.17)	0.97 (0.83-1.12)	1.32 (1.23-1.41)	0.94 (0.84-1.05)
**Puerto Rico**	0.99 (0.97-1.01)	0.95 (0.91-1.00)	0.99 (0.98-1.01)	1.01 (0.96-1.06)	1.00 (0.95-1.04)	1.09 (1.03-1.15)	1.42 (1.19-1.71)	1.11 (1.04-1.18)	0.95 (0.89-1.02)	1.10 (1.07-1.14)	1.04 (0.99-1.09)
**Peru urban**	0.97 (0.92-1.02)	1.00 (0.89-1.11)	1.05 (0.99-1.12)	0.95 (0.85-1.07)	0.94 (0.84-1.05)	1.28 (1.14-1.44)	1.69 (1.39-2.05)	1.26 (1.07-1.48)	0.89 (0.72-1.09)	1.38 (1.07-1.48)	1.21 (1.03-1.41)
**Peru rural**	1.03 (0.91-1.16)	0.93 (0.70-1.22)	0.98 (0.85-1.13)	1.07 (0.80-1.43)	1.33 (0.99-1.80)	1.04 (0.78-1.38)	1.20 (0.87-1.65)	1.64 (1.09-2.47)	1.12 (0.72-1.75)	1.53 (1.25-1.87)	1.38 (0.97-1.96)
**Venezuela**	1.02 (0.99-1.06)	0.88 (0.81-0.96)	1.00 (0.96-1.04)	0.99 (0.91-1.07)	0.96 (0.89-1.04)	1.06 (0.98-1.14)	1.11 (1.04-1.19)	1.14 (1.03-1.27)	0.86 (0.73-1.00)	1.19 (1.14-1.25)	0.98 (0.89-1.09)
**Mexico urban**	1.04 (1.00-1.08)	0.89 (0.82-0.98)	1.02 (0.99-1.06)	1.06 (0.97-1.15)	1.03 (0.95-1.11)	0.95 (0.87-1.03)	1.19 (1.10-1.29)	1.03 (0.89-1.20)	0.92 (0.80-1.06)	1.12 (1.06-1.17)	1.10 (0.89-1.13)
**Mexico rural**	0.97 (0.93-1.02)	0.87 (0.79-0.96)	1.02 (0.96-1.08)	0.96 (0.87-1.05)	1.03 (0.94-1.13)	1.02 (0.92-1.12)	1.22 (1.12-1.34)	1.01 (0.82-1.25)	0.93 (0.78-1.12)	1.07 (1.01-1.14)	1.01 (0.89-1.09)
**China urban**	0.98 (0.91-1.05)	0.93 (0.80-1.08)	1.04 (0.98-1.10)	0.98 (0.84-1.16)	1.08 (0.94-1.24)	1.34 (1.16-1.55)	2.02 (1.36-3.00)	0.70 (0.16-3.00)	0.93 (0.72-1.20)	1.88 (1.70-2.08)	0.90 (0.72-1.14)
**China rural**	0.79 (0.60-1.03)	1.03 (0.62-1.73)	0.86 (0.64-1.16)	1.00 (0.60-1.65)	1.22 (0.66-2.25)	0.57 (0.32-1.03)	1.63 (0.84-3.16)	Too few exposed	1.62 (0.72-3.68)	3.02 (2.19-4.17)	2.44 (1.08-5.46)
**India urban**	1.00 (0.95-1.05)	0.86 (0.76-0.96)	1.02 (0.98-1.07)	1.46 (1.29-1.67)	1.14 (1.00-1.30)	1.44 (1.28-1.62)	1.02 (0.70-1.50)	1.20 (0.97-1.49)	0.93 (0.74-1.16)	1.51 (1.39-1.63)	1.15 (1.02-1.29)
**India rural**	0.95 (0.90-0.99)	1.04 (0.92-1.17)	1.03 (0.97-1.09)	1.00 (0.89-1.12)	0.97 (0.89-1.07)	0.93 (0.84-1.02)	Too few exposed	1.57 (1.47-1.67)	0.93 (0.82-1.07)	1.19 (1.13-1.26)	0.95 (0.85-1.07)
**Nigeria**	0.99 (0.91-1.08)	1.04 (0.84-1.29)	1.14 (1.06-1.24)	0.89 (0.72-1.11)	Not assessed	Not assessed	Too few exposed	2.32 (1.56-3.34)	1.16 (0.88-1.53)	2.00 (1.70-2.36)	0.94 (0.66-1.33)

**Meta-analytical pooled effect**	0.99 (0.98-1.00)	0.93 (0.91-0.96)	1.03 † (1.01-1.05)	1.03 † (0.98-1.09)	1.01 (0.99-1.04)	1.08 † (1.00-1.17)	1.27 † (1.16-1.38)	1.21† (1.07-1.38)	0.93 (0.90-0.97)	1.37 † (1.26-1.49)	1.02 † (0.96-1.09)
**Cochrane Q **[df] (p value)	18.1 [12] (0.11)	13.4 [12] (0.34)	27.9 [12] (0.006)	38.5 [12] (< 0.001)	13.9 [11] (0.24)	81.4 [11] (< 0.001)	28.8 [9] (0.001)	100.0 [11] (< 0.001)	7.8 [12] (0.80)	244.0 [12] (< 0.001)	38.3 [12] (< 0.001)
**I^2 ^Higgins**, % (95% CI)	45 (0 - 73)	11 (0-51)	0 (0-60)	72 (49-85)	26 (0-64)	88 (80-92)	60 (23-80)	88 (0-93)	0 (0-60)	94 (91-96)	73 (51-85)

*All estimates mutually adjusted for all other variables in model 1

† Adjusted for all variables in model 1

† Random effects pooled meta-analysis

At the centre level, the proportion of consultations requiring out-of-pocket expenses was inversely associated with health insurance coverage (-0.59, p = 0.04) and pension coverage (-0.51, p = 0.07). The ecological associations with the prevalence of health service use were stronger for the proportion of consultations requiring out-of-pocket expenses (-0.50, p = 0.09) than for health insurance coverage (0.07, p = 0.83) and pension coverage (0.18, p = 0.57).

## Discussion

We carried out catchment area surveys of representative samples of older people in six Latin American countries (Cuba, Dominican Republic, Puerto Rico, Venezuela, Mexico and Peru), India, China and Nigeria. In all we completed 17,944 interviews with a high response rate in most sites. Our eight urban and five rural catchment area sites encompassed wide variation in age distributions, prevailing economic circumstances, levels of education, and pension and health insurance coverage. Our data adds considerably to current understanding of patterns of service utilisation among older people in LMIC, with respect to the range of services assessed (using a standard protocol in all sites), the detailed assessment of health status, and the comprehensive assessment of predisposing and enabling variables, including the impact of health insurance coverage. There are potential limitations, particularly with respect to the self-reported data on health service use. Utilization of healthcare (in practice) is not the same as access to healthcare (in principle), which may be the more relevant construct with respect to issues of equity [7]. Information systems in most LMIC are inadequate for routinely recorded data to be used. Studies have shown that self-reported health service use may be biased by a slight tendency towards underreporting, and that measures are most accurate for recent episodes of medical care [27]. We used data on recent use (the three months preceding the interview). Misclassification is likely to have been random with respect to correlates of healthcare utilisation, biasing any associations towards the null. This may not have been the case for dementia since those with the condition might have been less likely to recall service use, and the family informant that was relied upon under such circumstances may not have always been aware. Finally, while the common research protocols and demonstrable cross-cultural validity of our methods allow direct comparisons to be made between samples from the different countries surveyed, our findings should be generalized with caution and only to populations similar to those that we focused upon. Certain of the local health systems could not be considered to be typical of rural and urban settings in the country concerned, particularly in India, where Voluntary Health Services in urban Chennai and the Christian Medical College in rural Vellore operate as charitable non-governmental service providers running comprehensive primary care, inpatient and outpatient hospital services free of charge to those who cannot afford to subscribe or pay.

We found that the overall levels of use of any community healthcare services in the past three months varied very widely among sites, with little of this variation explained by compositional differences in age, sex and the prevalence of physical, mental and cognitive disorders. In almost all of the settings studied, the healthcare systems were mixed, with private providers played an important role in all countries other than Cuba and China, particularly in the Dominican Republic, Puerto Rico, Venezuela and India, where, other than in Puerto Rico, out-of-pocket payments were the rule. Out-of-pocket payments were also required for almost all government primary care and hospital outpatient consultations in China, and for a high proportion of these consultations in rural Peru and Nigeria. In China, the proportion of participants claiming to have health insurance does not provide a true picture of the extent of cover provided. In urban China, there are two employee-based health insurance schemes, one for government and the other for public and private company employees. In rural China, the government contributes to a common fund covering healthcare costs but only proportionate to the amount contributed locally. This rural Cooperative Medical System is ineffective, and the cover provided in cities is patchy; none of the schemes has kept pace with the sharp increases in charges under the new fee-for-service system [28], and the majority of healthcare costs are covered by out-of-pocket payments [29]. The extent of cover provided in other settings will have varied considerably among individuals claiming to have private health insurance. One of the limitations of our assessment of the effect of health insurance on service use is that we have individual level data only on whether or individuals have private health insurance, and not on the extent of the cover provided to the individual. Although causality is hard to demonstrate, the extent of out-of-pocket payments, coupled with the extent of pension and health insurance coverage, provide a parsimonious explanation for the observed variation in the overall levels of healthcare utilisation between sites, as well as for the type of service preferentially consulted within sites. It cannot however account for the relative unpopularity of low cost government primary care services in the Dominican Republic and in India, compared to private healthcare providers, particularly given the relatively low levels of private health insurance coverage in these sites.

Need for healthcare, as indicated by the presence of chronic health conditions, was the main determinant of use of healthcare services in all sites. However, while number of limiting physical impairments and ICD-10 depression were associated with the use of healthcare services in most sites, dementia was consistently inversely associated. This observation is consistent with the earlier impression, principally from qualitative research in India, of low levels of awareness of dementia as a medical condition, limited help-seeking, and generally unhelpful responses from healthcare services [30,31]. It will be important in the future to explore factors associated with help-seeking among people with dementia, particularly the role of disease severity and the presence of behavioural or psychological symptoms. The effective rationing of community healthcare to those with the most evident physical or psychological morbidity was particularly apparent in those sites such as rural China and Nigeria, with generally low levels of use of healthcare services. It was not possible to determine whether demand or supply, or a combination of both, contributed to this pattern of usage.

Inequity was particularly evident in the positive association between educational level and use of healthcare services, and the strong effect of health insurance cover on use of community healthcare services at least in those sites where out-of-pocket expenses were common, and where private health insurance was an important component of healthcare financing. In this context, the heterogeneous association between household assets and use of healthcare services was puzzling. For countries such as Cuba, where healthcare is free at the point of delivery, it makes sense that educational level rather than socioeconomic position is associated with increased healthcare utilisation particularly of prevention and promotion services, and through better adherence to chronic disease management protocols. In some other settings, it may be that household assets, an index of accumulated wealth, may be less salient than regular personal income to meet out-of-pocket expenses. We found no consistent evidence for ageism in the demand for or delivery of healthcare services, although older people were significantly less likely to use healthcare services in Cuba and rural India, after adjusting for health status and other variables, with a strong trend in that direction in rural China. There was also little evidence for the exclusion of those with disabilities, at least as regards those with restricted mobility. Of course, one of the likely mechanisms for the under usage of healthcare services by people with dementia may be the impact of cognitive disability on help seeking, compounded by the lack of outreach services to meet the special needs of this group [32]. The finding that women are more likely than men to access community healthcare services is now also conclusively demonstrated for older people in LMIC. We have previously reported that the apparent excess disability among women in our surveys may be accounted for by selective underreporting by men [32]. Studies from LMIC suggest that men have a higher mortality in late-life [33,34], and in our survey, as in others, are much more likely to be admitted acutely to hospital. Attention should therefore be directed towards encouraging timely help seeking, detection, treatment and control of chronic diseases and their risk factors among men.

## Conclusions

The most striking finding from this international survey is the very wide variation in the proportions of older people accessing community healthcare services in the past three months between sites, even after accounting for compositional differences in age and sex and the prevalence of chronic health conditions. Aside from cultural differences in help-seeking, this seems likely to be accounted for by stark differences in the extent of out-of-pocket expenses, and the ability of older people and their families to afford them. Health service utilisation was highest in Puerto Rico, where most older people were covered by Medicare, and lowest in China, where out-of-pocket costs were almost universal; across the 13 sites, the ecological correlation between the proportion of consultations requiring out-of-pocket costs and the prevalence of health service use was large (-0.50) and of borderline statistical significance (p = 0.09). Out-of-pocket costs are linked directly to government policies on the financing and reimbursement of healthcare. Intergovernmental charters, conventions and plans stress the importance of equity in the provision of healthcare to older people, and provide a framework of rights and responsibilities which, once ratified, are binding in international law. Data from epidemiological studies can remind governments of their obligations, provide evidence of the extent to which these have, or have not been met, and hence assist stakeholders in holding governments to account. The results of our surveys suggest that health systems that rely to a significant extent upon out-of-pocket payments for financing basic medical services risk excluding three overlapping groups; the poorest older people in society, those that lack a secure regular income, and those without health insurance. The strong independent effect of education on healthcare utilisation suggests that more may need to be done to promote demand for healthcare, even in those settings, such as Cuba, where economic barriers are less of a problem. Finally, we should note that achieving equity in access to healthcare is not the same as achieving equity in health [7]. While this may be one important driver, the determinants of health inequalities are complex and operate across generations and throughout the life course.

## Competing interests

The 10/66 Dementia Research Group works closely with ADI, which is a non-profit federation of 77 Alzheimer associations around the world. ADI is committed to strengthening Alzheimer associations worldwide, raising awareness regarding dementia and Alzheimer's disease, and advocating for more and better services for people with dementia and their caregivers. Daisy Acosta is the currently the chairperson of ADI. ADI is supported in part by grants from GlaxoSmithKline, Novartis, Lundbeck, Pfizer, and Eisai.

## Authors' contributions

EA and MP jointly conducted the analyses and wrote the manuscript. All of the authors worked collectively to develop the protocols and methods described in this paper. MP leads the 10/66 Dementia Research Group and CPF acts as study co-ordinator with EA; JJLR (Cuba), DA (Dominican Republic), MG (Peru), AS (Venezuela), ALS (Mexico), KSJ (Vellore, India), JW (Chennai, India), and YH (China) are principal investigators responsible for the field work in their countries. ATJ (Chennai, India) and MGK (Cuba) were the project coordinators in their respective sites working with principal investigators in the coordination of the fieldwork. All other authors reviewed the report and provided further contributions and suggestions. All authors read and approved the final report.

## Pre-publication history

The pre-publication history for this paper can be accessed here:

http://www.biomedcentral.com/1472-6963/11/153/prepub
